# Association between immune-related adverse events and the prognosis of patients with gastric cancer treated with nivolumab: a meta-analysis

**DOI:** 10.3389/fonc.2024.1408755

**Published:** 2024-09-05

**Authors:** Ya-Jun Zhang, Qian-Yu Tian, Cai-E. Wang

**Affiliations:** Department of Pharmacy, The First Affiliated Hospital, and College of Clinical Medicine of Henan University of Science and Technology, Luoyang, China

**Keywords:** immune checkpoint inhibitors, nivolumab, immune-related adverse events, gastric cancer, meta-analysis

## Abstract

**Background:**

Nivolumab is an effective treatment option for advanced gastric cancer (GC). This study aimed to conduct a systematic review of existing literature to investigate the relationship between immune-related adverse events (irAEs) and the prognosis of patients with GC treated with nivolumab.

**Methods:**

We comprehensively searched four online literature databases: the Cochrane Central Register of Controlled Trials, PubMed, Embase, Web of Science, until 27 March 2024. The outcome measures of interest included: overall survival (OS), progression-free survival (PFS), hazard ratio (HR), median survival ratio (MSR), objective response rate (ORR), and disease control rate (DCR).

**Results:**

A total of six studies, including 393 patients, met the eligibility criteria. The OS (pooled hazard ratio [HR] = 0.4, 95% confidence interval [CI]: 0.3–0.6, *p* < 0.05) and PFS (pooled HR = 0.5, 95% CI: 0.4–0.6, *p* < 0.05) of patients with irAEs were significantly superior to individuals without irAEs. The MSR for OS and PFS were 2.5 (95% CI: 1.5-4.1, *p* < 0.05) and 2.8 (95% CI: 1.9–4.1, *p* < 0.05), respectively. Regarding the ORR and DCR, we found that the development of irAEs was significantly associated with higher rates: patients with irAEs had an ORR of 24.7% compared to 6.4% in those without irAEs (risk ratio [RR] = 2.6, *p* < 0.05), and a DCR of 86.0% compared to 30.3% in those without irAEs (RR = 3.2, *p* < 0.05).

**Conclusion:**

There appears to be a significant correlation between the development of irAEs and the better survival benefits with nivolumab in patients with GC.

**Systematic review registration:**

https://www.crd.york.ac.uk/prospero/, identifier CRD42022341396.

## 1 Introduction

Globally, gastric cancer (GC) ranks as the third leading cause of cancer-related mortality and the fifth most prevalent kind of cancer (1). The management of unresectable advanced or metastatic GC involves antiangiogenic therapy, chemotherapy and targeted therapy (2–5). Immune checkpoint inhibitors (ICIs) have evolved into the standard of care for many patients with advanced solid malignancies over the past decade. ICIs, which have shown remarkable potential as treatments for GC, primarily include programmed cell death ligand 1, programmed cell death protein 1 (PD-1), and cytotoxic T-lymphocyte antigen 4 inhibitors (6, 7). Nivolumab, a monoclonal antibody targeting PD-1, has demonstrated clinical activity and notable efficacy in treating GC. Patients with advanced GC who receive nivolumab monotherapy as a third or subsequent treatment show improved survival (8).

However, patients treated with ICIs such as nivolumab, sometimes experience unique adverse events known as immune-related adverse events (irAEs). IrAEs are the adverse events that may have an immunological cause and may call for the administration of immunosuppressive or endocrine medication. The most common irAEs have been observed in the skin, endocrine system, gastrointestinal tract, and pulmonary system (9).

Recently, growing evidence has suggested a potential correlation between the development of irAEs and ICIs efficacy in patients with non-small cell lung cancer (NSCLC) and melanoma (10, 11). However, limited data are available regarding this relationship in GC patients. Individual clinical studies have been unable to characterize this association comprehensively. This study conducted a meta-analysis to elucidate the correlation between irAEs and nivolumab efficacy in patients with GC.

## 2 Materials and methods

### 2.1 Study design

The Preferred Reporting Items for Systematic Reviews and Meta-Analyses and MOOSE criteria were used in this meta-analysis (12, 13). We registered the meta-analysis at the International Prospective Register of Systematic Reviews (number CRD42022341396).

### 2.2 Search strategy

We conducted a comprehensive search of four online literature databases: the Cochrane Central Register of Controlled Trials, PubMed, Embase, Web of Science, until 27 March 2024 without setting the start date. The primary search terms used were gastric cancer, irAEs, immune checkpoint inhibitors, PD-1, and nivolumab. The search was limited to articles in English. Additionally, we reviewed the articles and pertinent study bibliographies.

### 2.3 Inclusion criteria and exclusion criteria

To qualify for inclusion in this analysis, studies must meet all of the following criteria: (1) adult patients were diagnosed with GC definitively; (2) Studies evaluating nivolumab monotherapy; (3) Studies that reported the relationship between irAEs and nivolumab efficacy in GC; (4) enough data reported on clinical outcomes.

Studies will be excluded from consideration based on any of the following criteria: (1) research irrelevant to our subjects or lacking relevant information; (2) Studies evaluating combination therapy; (3) Studies that involved fewer than ten patients; (4) study lacking retrievable or published full texts; (5) repeated publications.

### 2.4 Data extraction and quality assessment

The extracted data includes the name of the first author, the year of publication, trial design, median age, gender (% male), median follow-up, type and grade of irAEs, the median overall survival (OS) and progression-free survival (PFS), as well as the hazard ratios (HRs) and 95% confidence intervals (CIs) of OS and PFS in patients with and without irAEs; and the objective response rate (ORR) and disease control rate (DCR). The primary outcome was the HRs and 95% CIs for OS and PFS. The secondary outcome was median survival ratio (MSR) for the median OS and PFS, and the risk ratio (RR) for ORR and DCR. In each of the six included studies, the patients were divided into two groups (irAE and non-irAE groups) based on occurrence of irAEs during nivolumab treatment. We referred to original studies to resolve any data discrepancies.

Using the modified Newcastle-Ottawa Scale (NOS), we conducted an evaluation of the methodological quality of the studies (13). This scale encompasses three key areas: the selection process of cohorts, the comparability between study groups, and the evaluation of the outcome of interest.

### 2.5 Statistical analysis

For meta-analysis, the strength of the correlation between irAE development and nivolumab efficacy was calculated using pooled HRs and MSRs. The RRs and 95% CIs were calculated for the ORR and DCR. Weighted averages were calculated for studies reporting the median OS, median PFS, ORR and DCR in patients with and without irAEs. Cochrane’s Q and I^2^ tests were used to check between-study heterogeneity (14). In the case of significant heterogeneity, the random effect model was employed, which was defined as I^2^ > 50% or *p* ≤ 0.05. To examine the sources of heterogeneity, we conducted sensitivity analyses and meta-regression. We employed both Egger’s and Begg’s tests to evaluate the possibility of publication bias. For statistical significance, a two-sided *p* value of 0.05 was used. Stata/SE version 15.1 was used to conduct all statistical analyses (Stata Corporation, USA).

## 3 Results

### 3.1 Selection of studies and patients’ characteristics

**Figure 1:**
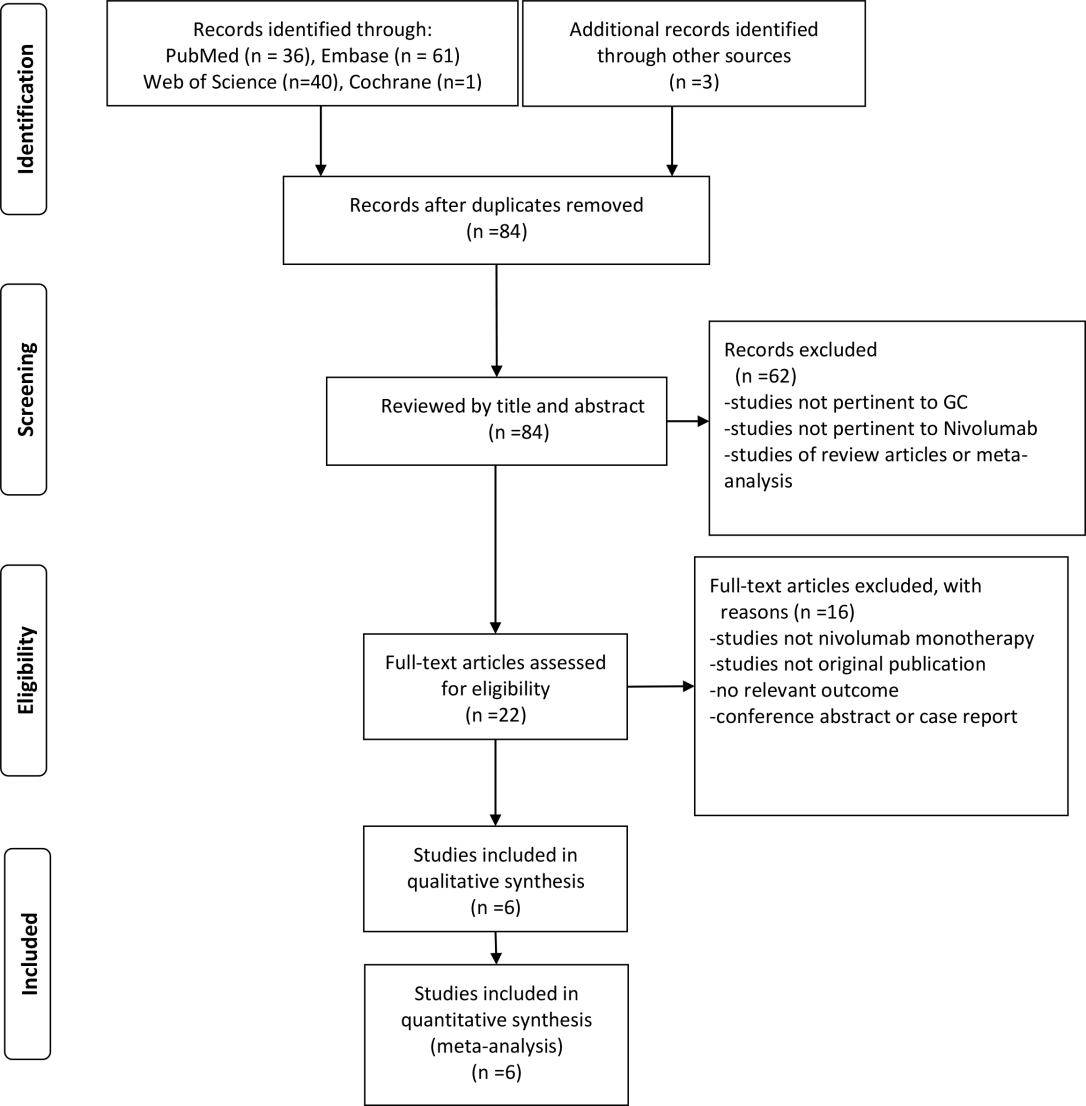
Flow diagram for study selection.

Our comprehensive search methodology identified 141 articles. After removing duplicates and conducting a preliminary evaluation using our predefined inclusion and exclusion criteria, we sought the full texts of the remaining records. Full texts were available for 22 articles, which were then subjected to a more rigorous evaluation. To ensure the highest quality and relevance, we applied an additional set of stringent inclusion and exclusion criteria to these 22 articles. This secondary screening process resulted in a final selection of 6 studies that fully met all our study requirements. These 6 studies, involving a total of 393 patients, were included in our final analysis (15–20). **Figure 1** shows the process of study selection of this meta-analysis.

Study characteristics are shown in **Table 1**. The included studies had sample sizes ranging from 29 to 110 patients (median n = 62). All studies were retrospective. All six studies reported the incidence of irAEs of any grades. The median follow-up was between 5.4 and 32 months.

**Table 1:** Characteristics of patients with GC in the included studies.

Studies	Study design	Line of therapy	Study size	Median Age (years)	Female (%)	Median Follow-up (months) (95%CI)	Median time to onset of irAEs (days)	Incidence of any grade irAEs (%)	Version of CTCAE
**Masuda, 2019** (15)	retrospective	NA	65	66 (35-83)	21.5	32 (10.8-34.5)	30.5 (3-407)	21.5	ver.4.03
**Namikawa, 2020** (16)	retrospective	NA	29	71 (49–86)	34.5	32	NA	34.5	ver.4.03
**Kono, 2021** (17)	retrospective	third	52	70 (58-76)	34.6	NA	63 (30-85)	25	ver.4.1
**Ishido, 2022** (18)	retrospective	third	59	71 (43–86)	23.7	5.9 (0.6-43.6)	57 (0-279)	32.2	ver.5.0
**Matsunaga, 2022** (19)	retrospective	NA	78	62 (38–88)	24.4	5.4 (2.7-18.1)	NA	19.2	ver.4.03
**Suematsu, 2022** (20)	retrospective	NA	110	NA	28.2	6.6 (0.6-35.6)	NA	20	ver.4.0

AE, adverse event; GC, gastric cancer; IRAES, immune-related adverse events; NA, not available; 95% CI, 95% confidence interval; CTCAE, Common Terminology Criteria for Adverse Events.

### 3.2 Quality assessment and bias

**Figure 2:**
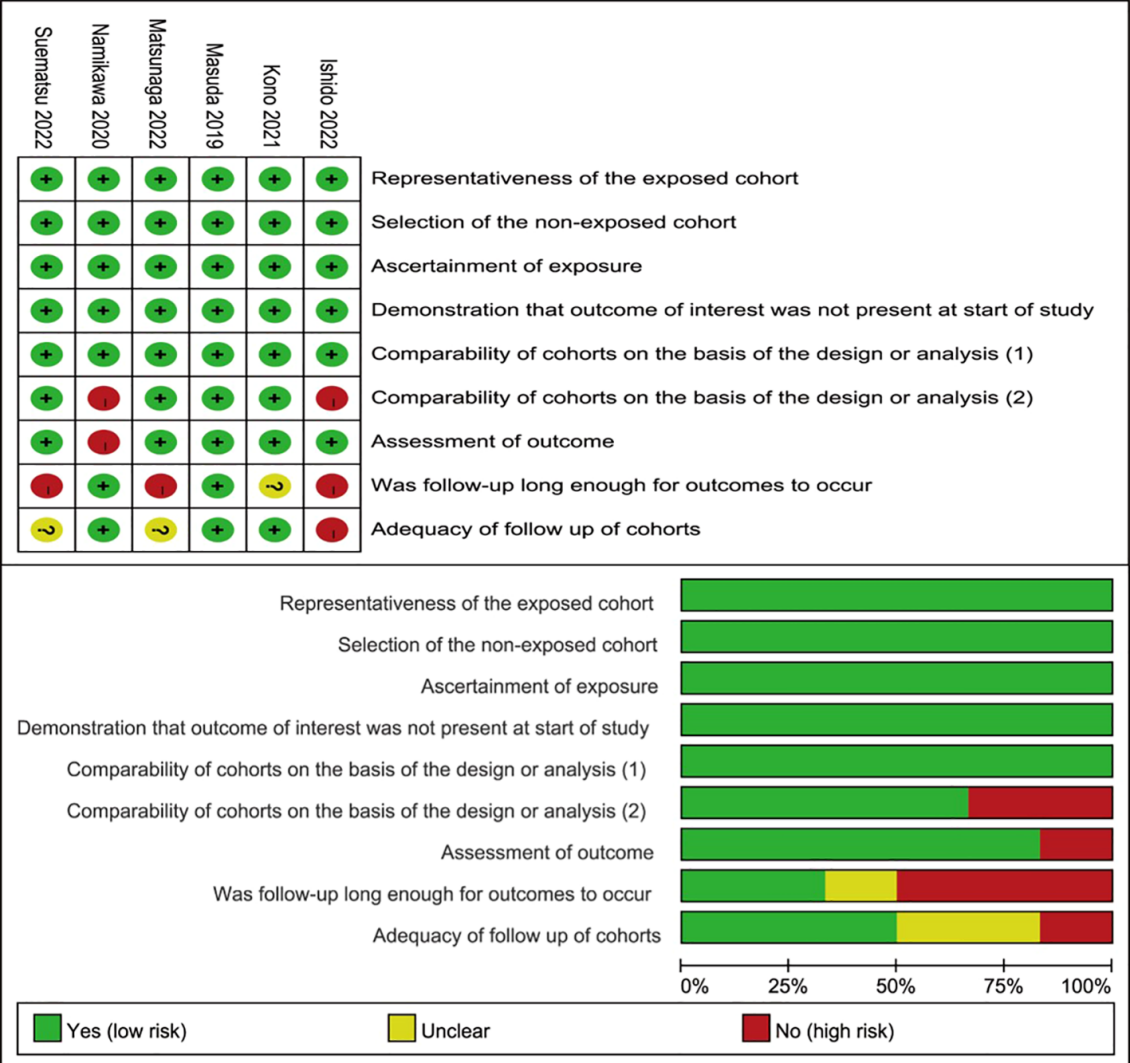
Risk of bias summary and risk of bias graph.

The six studies’ overall methodological quality was high (**Figure 2**). Considering that all the studies were retrospective, the quality of the included studies was suboptimal.

No evidence of significant publication bias was found in the main outcomes of this study (**Supplementary Table S1**). However, the statistical analysis of the PFS HRs suggested a possible publication bias.

### 3.3 Results of the meta-analysis

#### 3.3.1 OS and PFS

**Figure 3:**
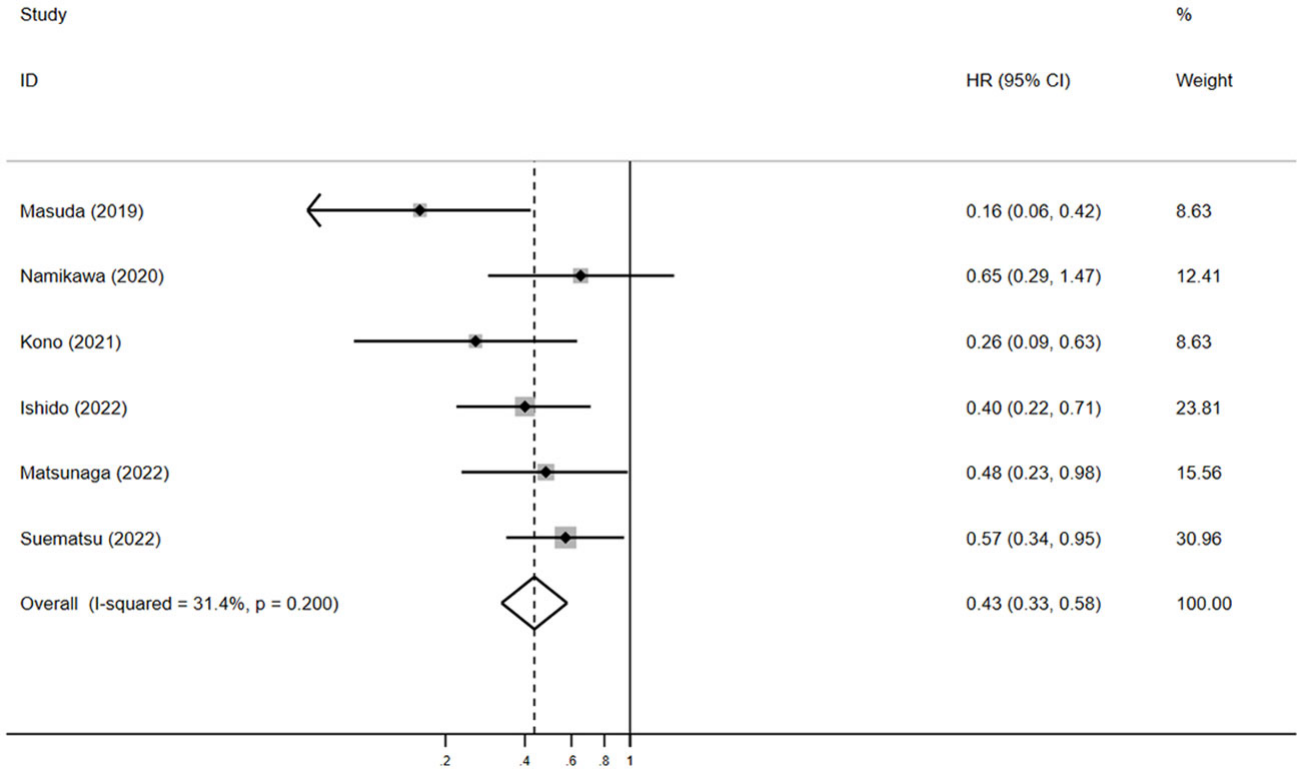
The pooled HR of OS for irAEs versus non-irAEs. HR, hazard ratio; IRAES, immune-related adverse events; OS, overall survival.

Six studies included OS data and six included PFS data (15–20). The outcomes of included studies were listed in **Table 2**. Five studies showed that patients with irAEs achieved longer OS, compared with those without irAEs (15, 17–20); however, Namikawa et al. found the median OS did not clearly differ between the irAE group and non-irAE group (6.2 months vs. 4.9 months, *p* = 0.3) (16). In this meta-analysis, pooled HR demonstrated a substantial positive association between the irAE group and favorable OS, in comparison to the non-irAE group (HR = 0.4, 95% CI: 0.3–0.6, *p* < 0.05) (**Figure 3**). The pooled HR for OS did not show any substantial heterogeneity (I^2^ = 31.4%, *p* = 0.2).

**Table 2:** Outcomes of the included studies of GC.

Studies	Analysis for OS and PFS (n)		Median OS (months)			HR for OS (95%CI)	Median PFS (months)			HR for PFS (95%CI)	Analysis for ORR and DCR (n)		Median ORR (%)		Median DCR (%)	
	irAE+	irAE-	irAE+	irAE-	P value		irAE+	irAE-	P value		irAE+	IrAE-	irAE+	irAE-	irAE+	irAE-
**Masuda, 2019** (15)	14	51	16.8 (4.4-NA)	3.2 (2.2-4.1)	<0.001	0.2 (0.1-0.4)	7.5 (3.6-11.5)	1.4 (1.2-1.6)	<0.001	0.1 (0.03-0.4)	11	34	27.3	0.0	100.0	23.5
**Namikawa, 2020** (16)	10	19	6.2	4.9	0.305	0.7 (0.3-1.5)	5.8	1.2	0.03	0.4 (0.20-0.9)	NA	NA	NA	NA	NA	NA
**Kono, 2021** (17)	13	39	NA (7.0-NA)	7.1 (5.0-8.4)	0.0036	0.3 (0.1–0.6)	4.7 (3.2-5.8)	1.6 (1.2-1.9)	0.008	0.4 (0.2-0.8)	8	21	25.0	14.0	88.0	24.0
**Ishido, 2022** (18)	19	40	19.1 (5.7-32.5)	4.7 (2.5-6.9)	0.002	0.4 (0.2-0.7)	3.1 (2.0-4.2)	1.7 (1.4-2.0)	0.008	0.5 (0.3-0.8)	NA	NA	NA	NA	NA	NA
**Matsunaga, 2022** (19)	15	63	9.4 (5.3-13.5)	5.8 (4.4-7.2)	0.041	0.5 (0.2-0.98)	4.9 (2.8-6.9)	2.6 (2.1-3.2)	0.018	0.5 (0.3-0.9)	15	63	26.7	9.5	86.7	42.9
**Suematsu, 2022** (20)	22	88	12.4	5.7	0.030	0.6 (0.3-0.95)	4.3	2.2	0.008	0.5 (0.3-0.9)	22	88	22.7	8.0	63.6	27.3

AE, adverse event; GC, gastric cancer; DCR, disease control rate; HR, hazard ratio; IRAES, immune-related adverse events; IRAE+, patients with immune-related adverse event; IRAE-, patients without immune-related adverse event; NA, not available; 95% CI, 95% confidence interval; ORR, objective response rate; OS, overall survival; PFS, progression-free survival.

**Figure 4:**
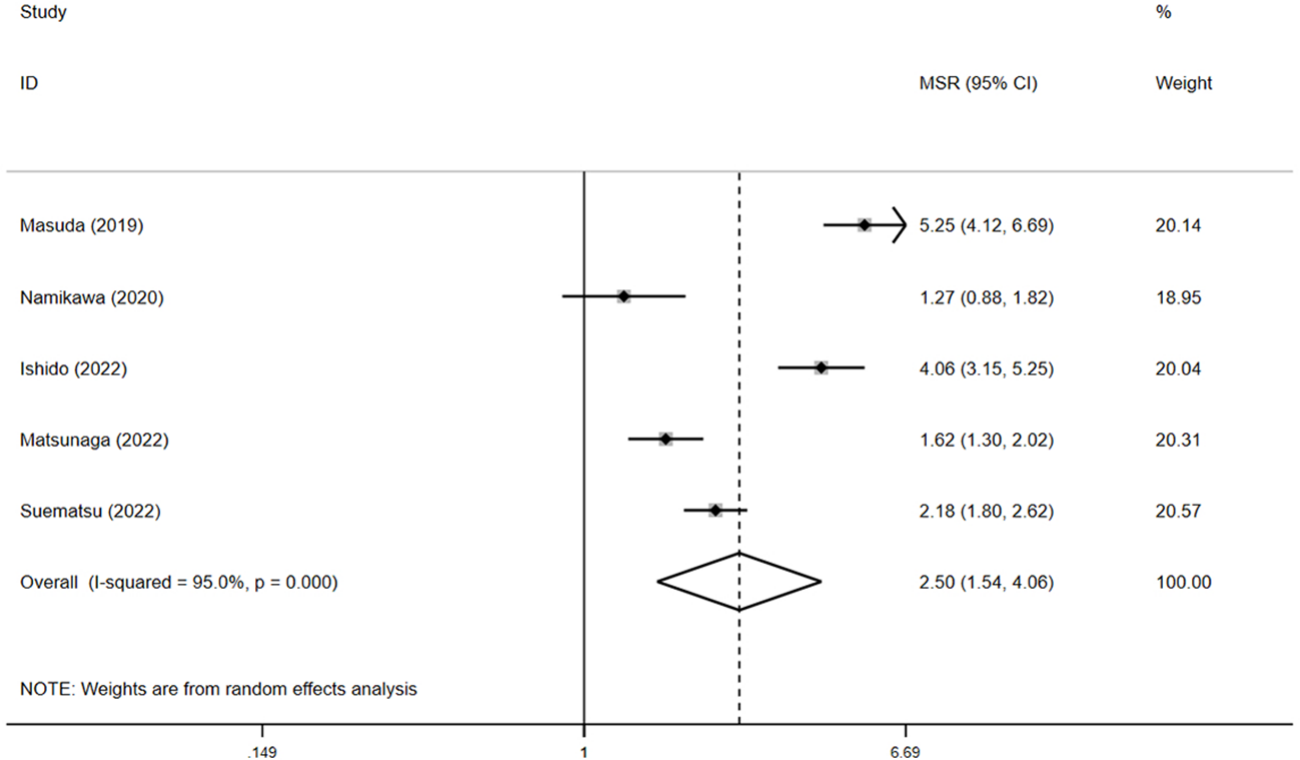
The pooled MSR of OS for irAEs versus non-irAEs. IRAES, immune-related adverse events; MSR, median survival ratio; OS, overall survival.

Among the six studies reporting OS data, four detailed the median OS and 95% CI (15, 17–19). The pooled median OS in the irAE group was 12.1 months (95% CI: 3.6–20.5, N = 2), compared with 4.6 months (95% CI: 3.9–5.3, N = 4) in the non-irAE group, showing a significant difference (pooled MSR = 2.5, 95% CI: 1.5–4.1, *p* < 0.05) (**Figure 4**).

**Figure 5:**
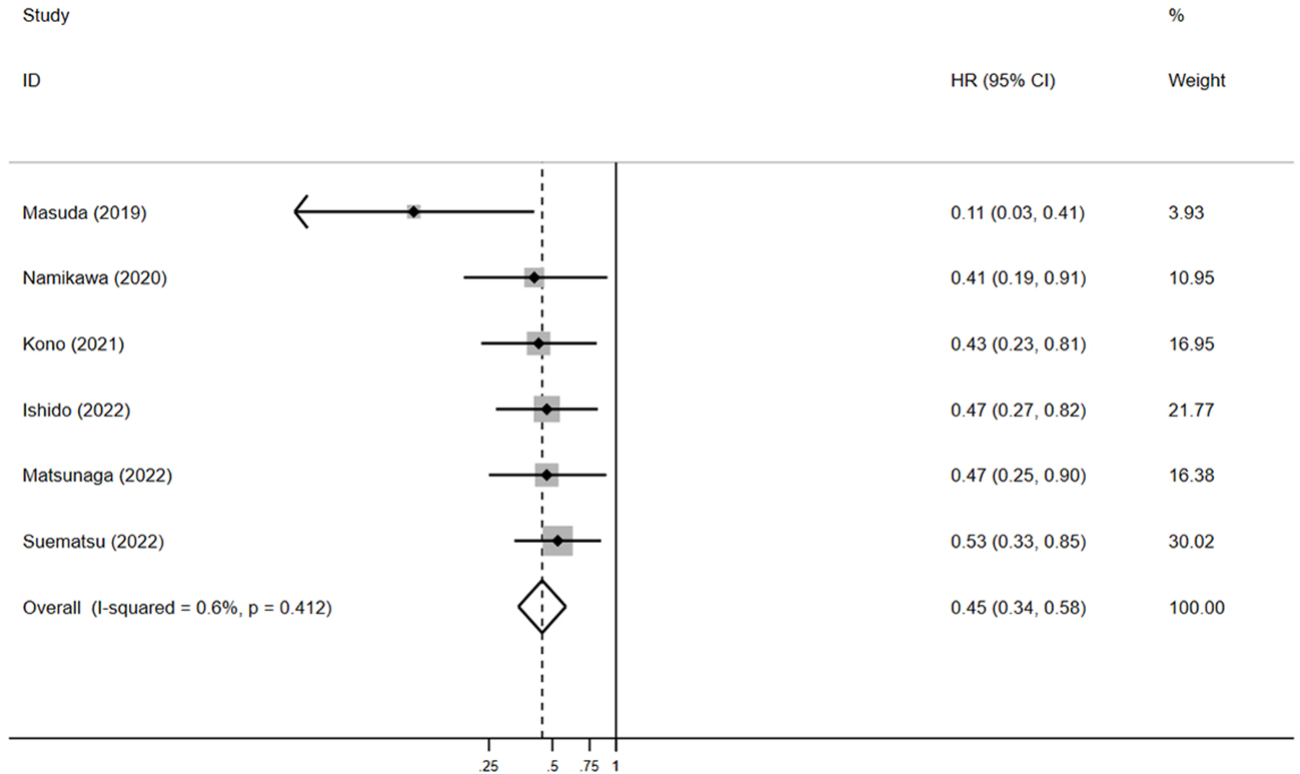
The pooled HR of PFS for irAEs versus non-irAEs. HR, hazard ratio; IRAES, immune-related adverse events; PFS, progression-free survival.

**Figure 6:**
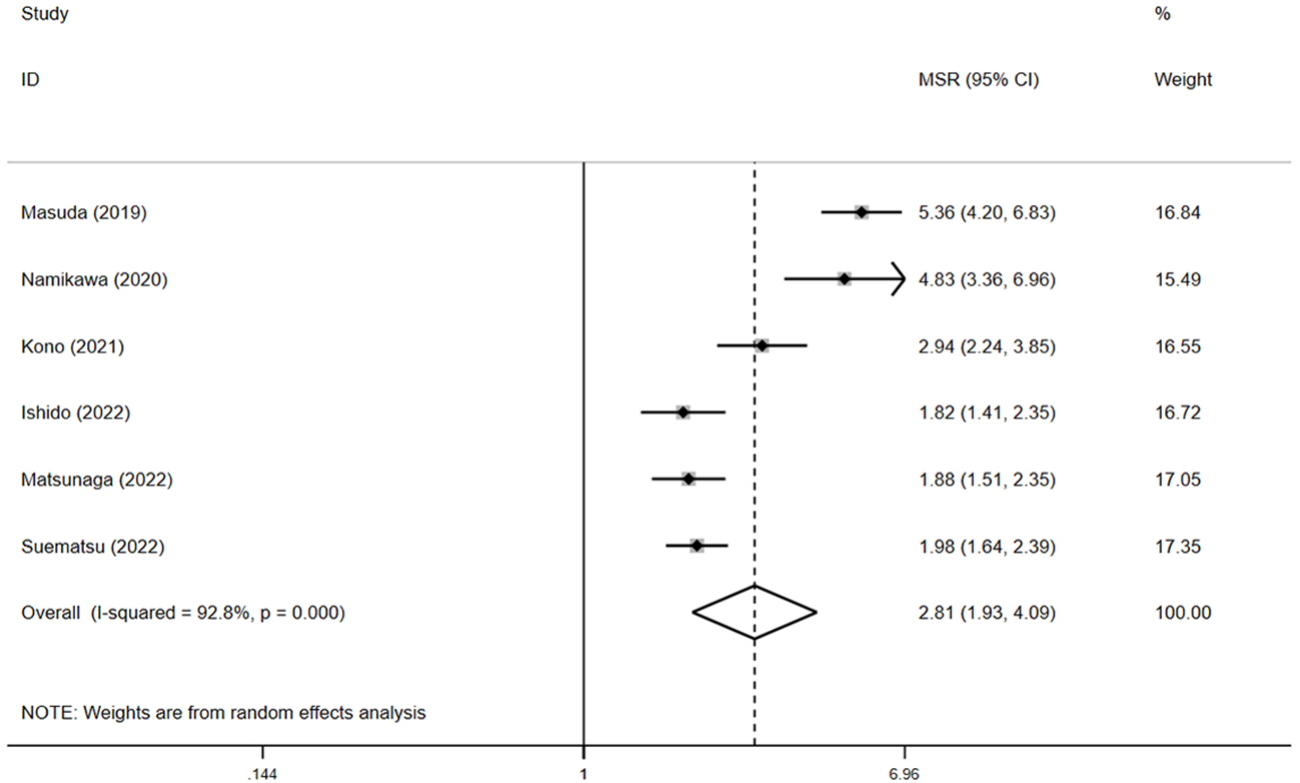
The pooled MSR of PFS for irAEs versus non-irAEs. IRAES, immune-related adverse events; MSR, median survival ratio; PFS, progression-free survival.

For PFS, pooled HR showed that patients experiencing irAEs had significantly better outcomes than those without irAEs (HR = 0.5, 95% CI: 0.4–0.6, *p* < 0.05) (**Figure 5**), with no significant heterogeneity in the studies (I² = 0.6%, *p* = 0.4). The pooled median PFS in the irAE group was 4.4 months (95% CI: 3.1–5.9, N = 4), compared to 1.6 months (95% CI: 1.4–1.7, N = 4) in the non-irAE group, indicating that PFS was significantly superior in patients with irAEs (pooled MSR = 2.8, 95% CI: 1.9–4.1, *p* < 0.05) (**Figure 6**).

#### 3.3.2 ORR and DCR

In the four studies reporting ORR data (15, 17, 19, 20), the pooled ORR in the irAE group was 24.7% (95% CI: 13.5%–37.7%, N=4) versus 6.4% (95% CI: 1.6%–13.4%, N=4) in the non-irAE group. In the irAE group, the ORR was significantly higher than that observed in the non-irAE group (RR=2.6, 95% CI: 2.0–3.3, *p* < 0.05) (**Supplementary Figure S1**).

In the four studies reporting DCR data (15, 17, 19, 20), the pooled DCR in irAE group was 86.0% (95% CI: 65.7%–98.8%, N=4) versus 30.3% (95% CI: 21.4%–39.9%, N=4) in the non-irAE group. Compared with patients without irAEs, individuals with irAEs were associated with significantly higher DCR (RR = 3.2, 95% CI: 1.7–6.2, *p* < 0.05) (**Supplementary Figure S2**).

#### 3.3.3 Grade of immune-related adverse events

Six trials recorded detailed irAEs of any grade (15–20). Overall, 93 (23.7%) of 393 patients developed one or more immune-related adverse events. The incidence of irAEs of any grade ranged from 19.2% to 34.5%. The most common irAEs of any grade were rash, diarrhea, and colitis.

Five studies reported whether grade 3 or higher irAEs occurred, and the rate varied from 1.8% to 15.3% in the different included studies (15, 17–20). Overall, 21 (5.8%) of 364 patients developed at least 1 grade 3 or higher irAEs. The most common grade 3 or higher irAEs were diarrhea and colitis.

In a study by Ishido et al., OS and PFS were compared in patients who had grade 1 or 2 toxicity after nivolumab treatment for GC versus those who had grade 3 or 4 toxicity (18). Patients experiencing low-grade irAEs (grades 1 or 2) generally showed better OS compared to those with severe irAEs (grades 3 or 4) (29.0 vs. 8.8 months, *p* = 0.1). However, there was no significant difference in PFS (*p* = 0.5).

### 3.4 Meta-regression and sensitivity analysis

The outcome of meta-regression analysis is presented in **Supplementary Table S2**. Sensitivity analyses revealed no significant sources of heterogeneity in any result. (**Supplementary Figures S3**-**S6**).

## 4 Discussion

ICI treatment is associated with certain types of adverse events due to the infiltration of highly activated CD4 and CD8 T cells, along with an increase in inflammatory cytokines in various normal tissues (21, 22). ICIs have recently been used to treat various malignancies. Patients diagnosed with GC who received ICIs did not demonstrate a statistically significant higher likelihood of experiencing any form of AEs linked to their therapy (23). According to Velasco, patients receiving ICIs are more likely to develop colitis, rashes, hypothyroidism, and pneumonitis (24). Another study found a similar rise in irAEs in ICIs and comprised 22 RCTs with solid organ malignancies (25). To date, whether the development of irAEs is related to the treatment of ICIs remains controversial. Herein, we conducted a meta-analysis that included data from six trials focusing on the correlation between the occurrence of irAEs and nivolumab efficacy in GC.

Our results demonstrated that patients with irAEs experienced a significantly longer OS and PFS compared with patients without irAEs. In addition, the pooled ORR and DCR in the irAE group were obviously higher than in the non-irAE group. Therefore, the presence of irAEs was strongly linked to improved effectiveness of nivolumab in treating GC. The most common grades of irAEs were rash, diarrhea and colitis. The most common grade 3 or higher irAEs were diarrhea and colitis. Our analyses are consistent with previous observations that irAEs have the ability to predict the response to nivolumab. The development of irAEs was associated with improved clinical outcomes. However, irAEs can sometimes be harmful, and severe irAEs may lead to permanent discontinuation of ICIs. Treatment interruptions due to severe irAEs are often associated with poorer survival outcomes compared to those who continue receiving ICI therapy (26). This association makes it critical to strengthen the monitoring of irAEs, and administer appropriate interventions for irAEs to guarantee uninterrupted administration of nivolumab and improve the patient’s long-term outlook. As the use of nivolumab continues to expand, timely identification of relevant symptoms and indications might assist doctors in formulating suitable approaches to manage these irAEs, thus minimizing the harmful effects of nivolumab and optimizing the treatment duration.

However, what irAEs-specific factors (such as severity, timing of onset or therapeutic intervention) play a prominent role in increasing survival remain unclear (27). Ishido et al. reported that the severity of irAEs is associated with survival and that low-grade irAEs may have improved OS (18). However, the types of irAEs associated with survival are not well-known. Yamamoto et al. reported no significant differences between irAEs associated with immune-related liver dysfunction and survival (28). Given the lead-time bias resulting from the time it takes for irAEs to occur, Masuda et al. conducted 8- and 12-week landmark analyses in GC and obtained equivalent survival results (15). In meta-regression analyses of irAEs according to median time to onset of irAEs, our study found no significant lead-time bias. Further analyses with larger sample sizes are required to explore the underlying mechanism and clinical significance.

Our study had several advantages. First, this study represents the initial meta-analysis of clinical trials providing comprehensive insights into irAEs and the effectiveness of nivolumab in patients with GC. Previous meta-analyses mainly focused on this relationship in melanoma and NSCLC (29, 30). Second, we conducted a thorough examination of all major clinical outcomes (OS, PFS, MSR, ORR and DCR) to elucidate the relationship between the occurrence of irAEs and the effectiveness of nivolumab in patients with GC. Third, to investigate the causes of heterogeneity more thoroughly, we conducted numerous sets of regression analyses of potentially linked factors. No significant heterogeneity was observed in the pooling of primary outcomes in our study, which may be attributed to the highly similar characteristics of the included populations.

Our study also has the following limitations. First, all data collected were from retrospective studies and not from prospective clinical trials, which may have led to information bias. Second, our study included a relatively small number of studies due to the limited availability of comprehensive prospective studies eligible for inclusion. Third, while one study provided valuable insight into the correlation between the severity of adverse events and nivolumab response, most of the included studies did not offer sufficient detailed data on this topic. This limitation precludes a comprehensive analysis across all studies. Future research with more extensive data on the correlation between adverse event grades and nivolumab response could provide deeper insights into this association. Finally, a publication bias for PFS was detected based on statistical analysis. Like all systematic reviews that rely on published literature, there is a risk of publication bias, particularly in studies with small sample sizes, such as in this meta-analysis. Thus, larger multicenter clinical trials are warranted to validate the findings of this study.

### 4.1 Conclusion

This meta-analysis indicated that the development of irAEs is associated with improved survival outcomes for nivolumab in patients with GC, which clinicians can reference to guide clinical treatment. To identify the patients most likely to benefit from nivolumab treatment for GC, further research and testing in a larger population are needed.

## Data Availability

The raw data supporting the conclusions of this article will be made available by the authors, without undue reservation.

## Supplementary material

Supplementary Figure 1 RR of DCR for irAEs versus non-irAEs.

Supplementary Figure 2 RR of ORR for irAEs versus non-irAEs.

Supplementary Figure 3 Sensitivity analyses for the pooled HR of OS, conducted by repeating the analyses with exclusion of studies one by one.

Supplementary Figure 4 Sensitivity analyses for the pooled MSR of OS, conducted by repeating the analyses with exclusion of studies one by one.

Supplementary Figure 5 Sensitivity analyses for the pooled HR of PFS, conducted by repeating the analyses with exclusion of studies one by one.

Supplementary Figure 6 Sensitivity analyses for the pooled MSR of PFS, conducted by repeating the analyses with exclusion of studies one by one.
