# Supplementary figures and images for: Association between immune-related adverse events and the prognosis of patients with gastric cancer treated with nivolumab: a meta-analysis
Source: Front Oncol. 2024 Sep 5;14:1408755. doi: 10.3389/fonc.2024.1408755 (PMC11410568; doi:10.3389/fonc.2024.1408755)

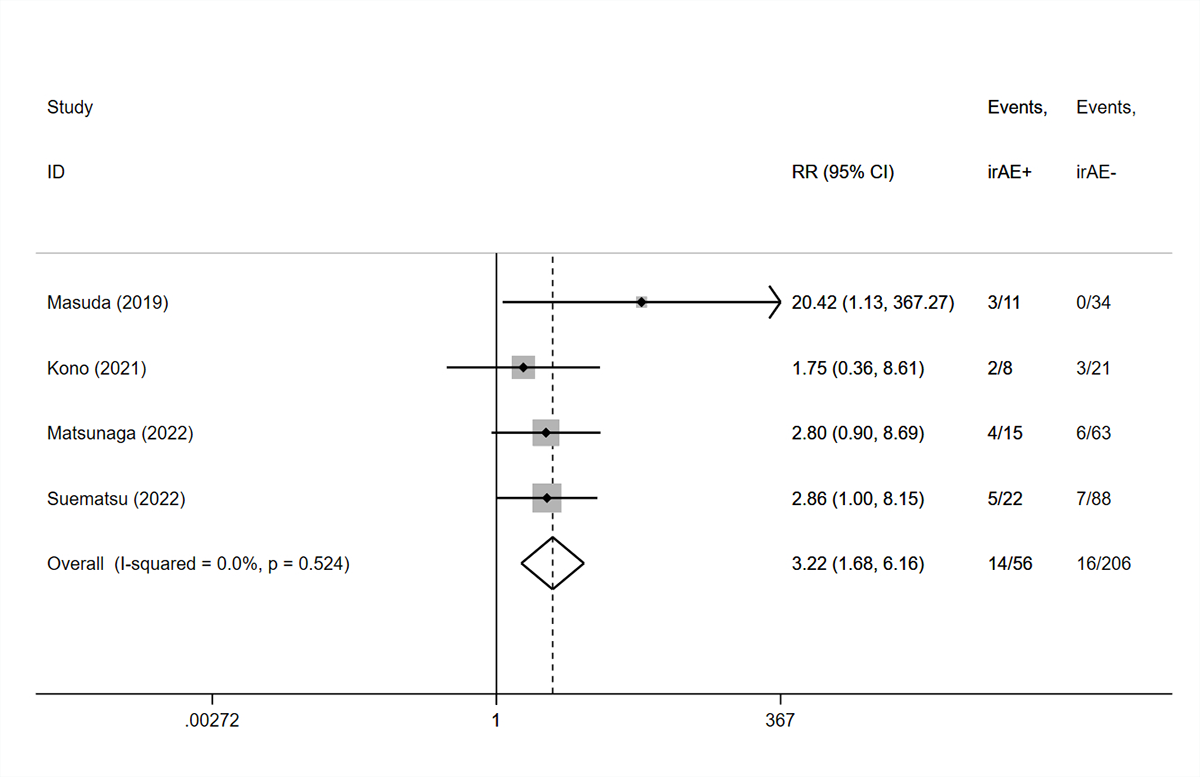

Supplement: Supplementary Figure 1 — RR of DCR for irAEs versus non-irAEs. [file Image1.tif]

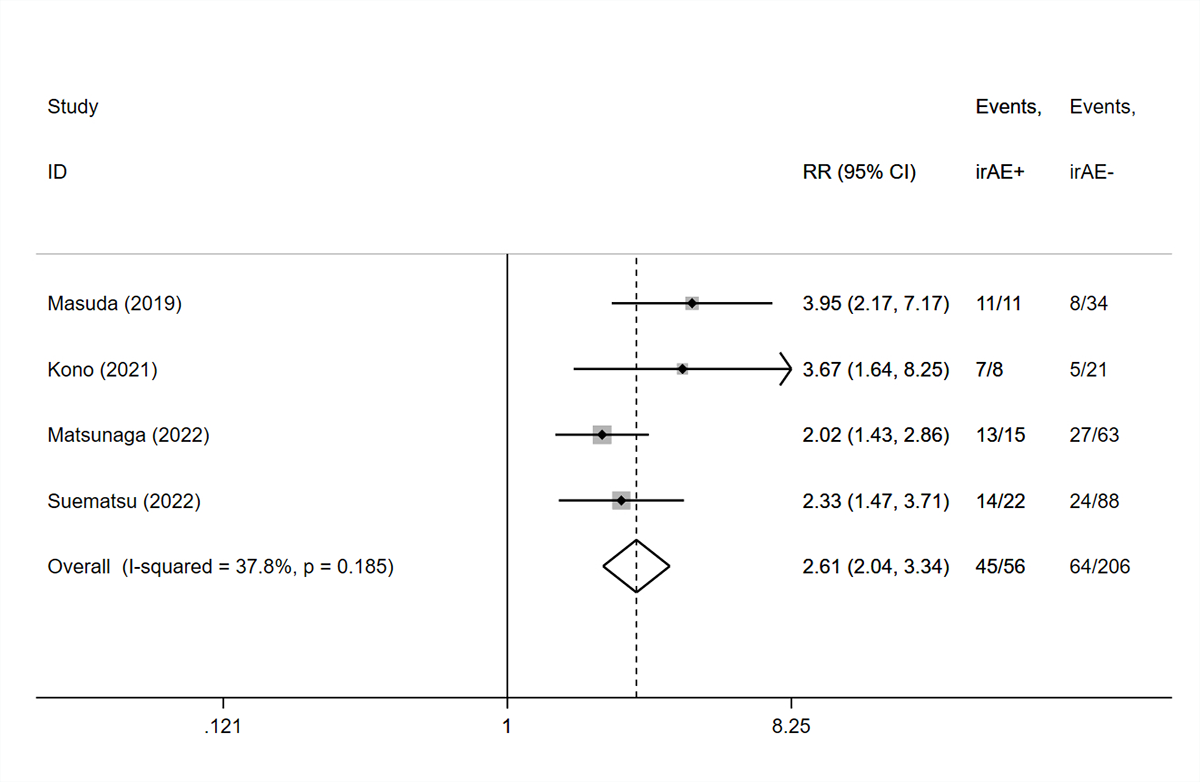

Supplement: Supplementary Figure 2 — RR of ORR for irAEs versus non-irAEs. [file Image2.tif]

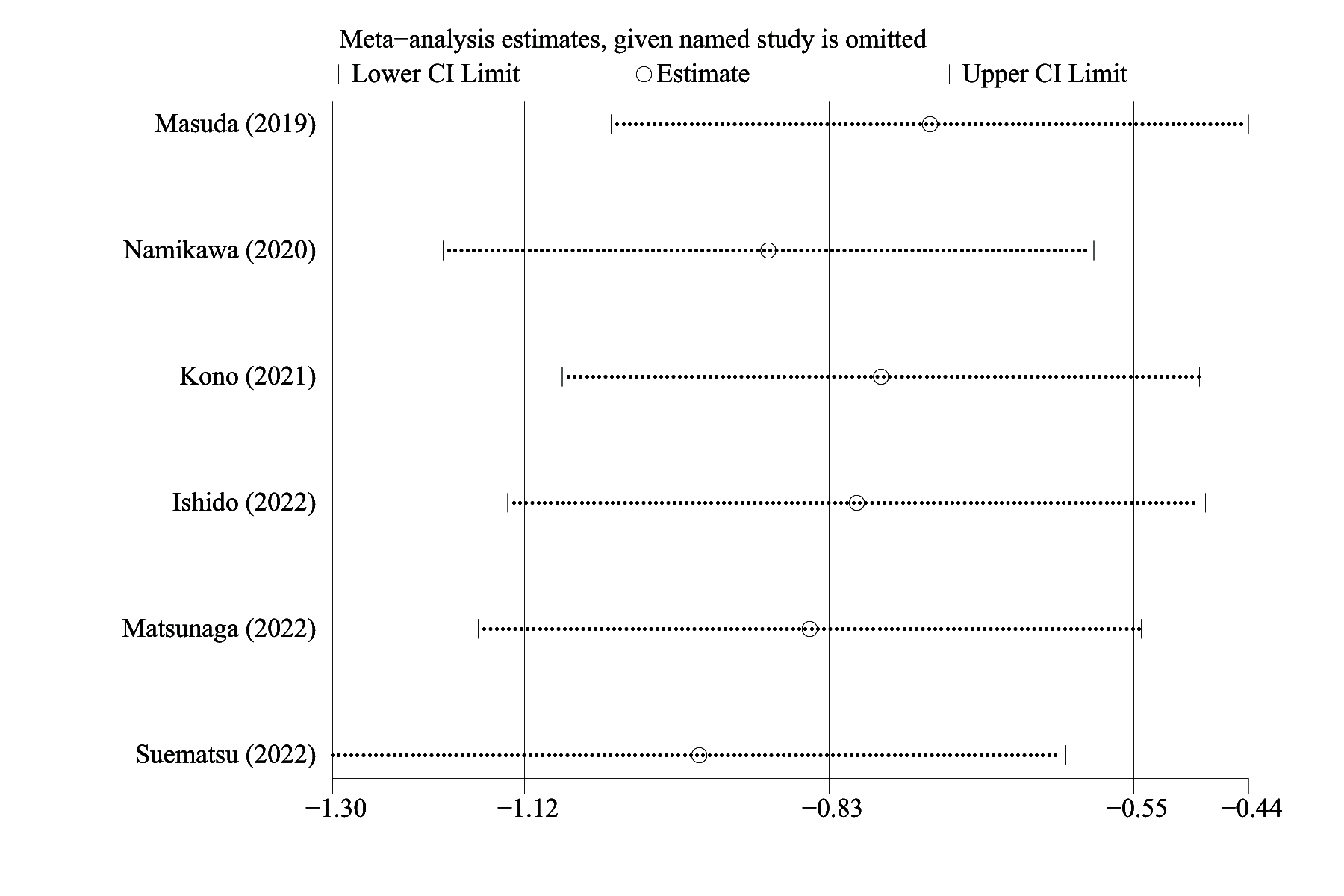

Supplement: Supplementary Figure 3 — Sensitivity analyses for the pooled HR of OS, conducted by repeating the analyses with exclusion of studies one by one. [file Image3.tif]

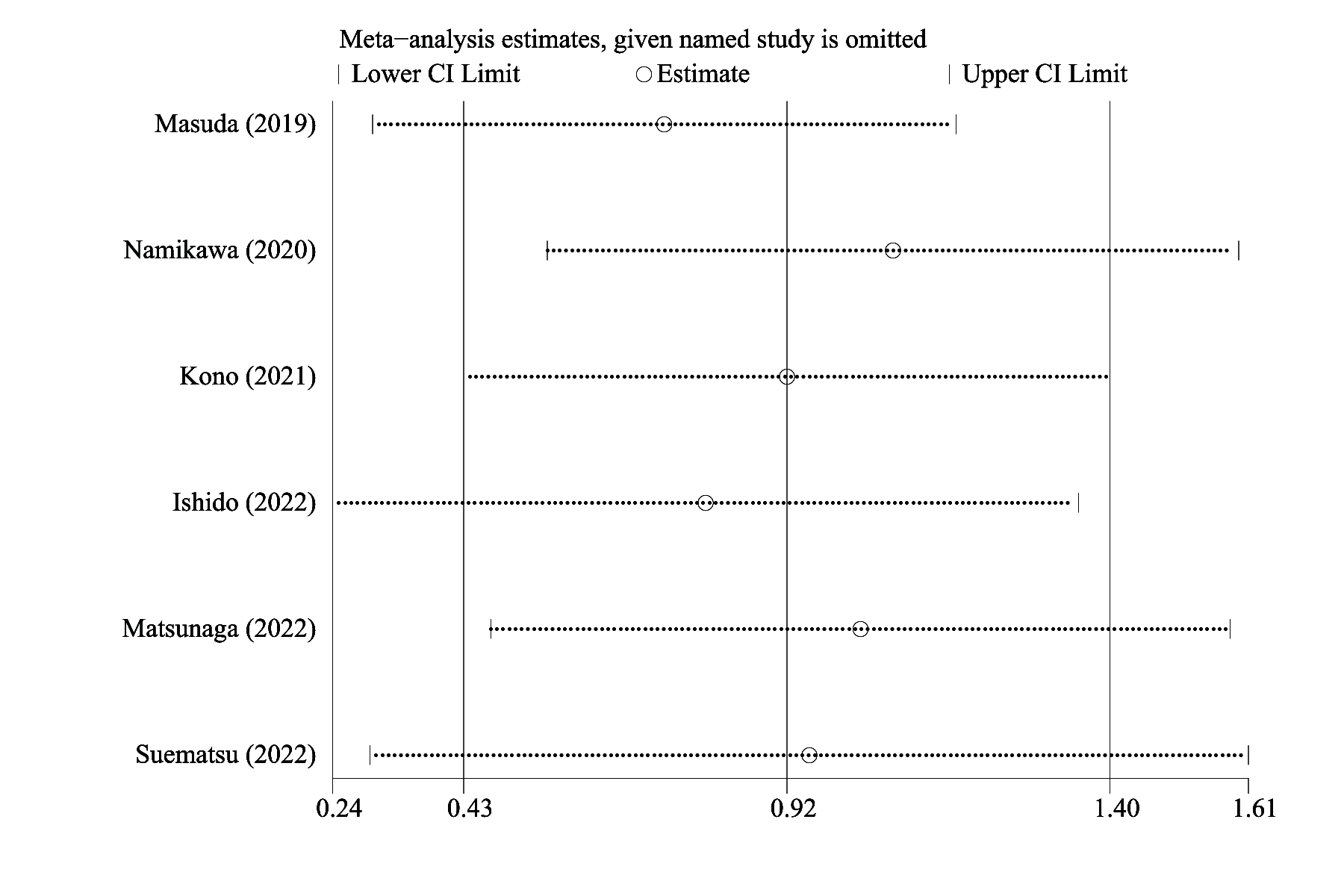

Supplement: Supplementary Figure 4 — Sensitivity analyses for the pooled MSR of OS, conducted by repeating the analyses with exclusion of studies one by one. [file Image4.tif]

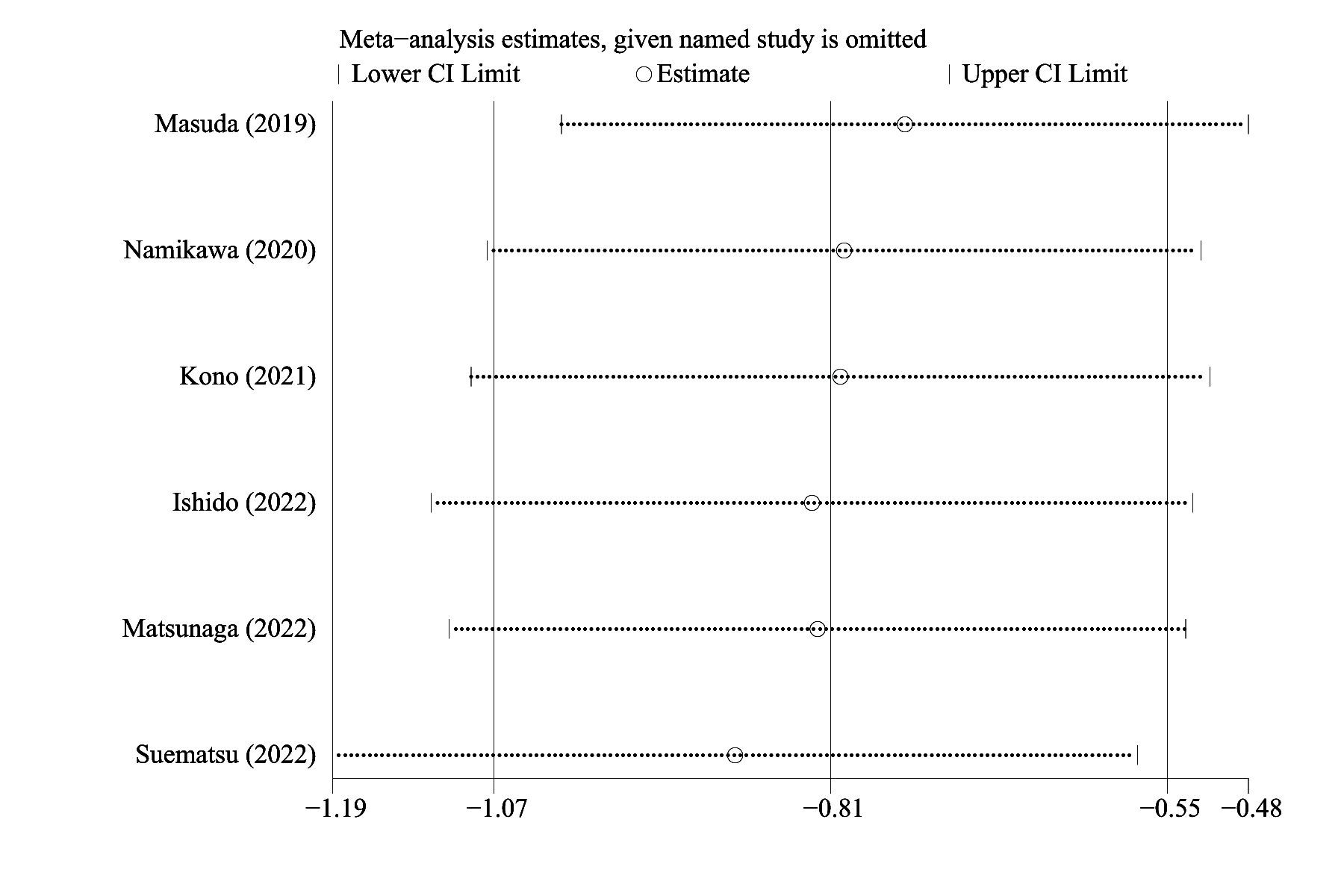

Supplement: Supplementary Figure 5 — Sensitivity analyses for the pooled HR of PFS, conducted by repeating the analyses with exclusion of studies one by one. [file Image5.tif]

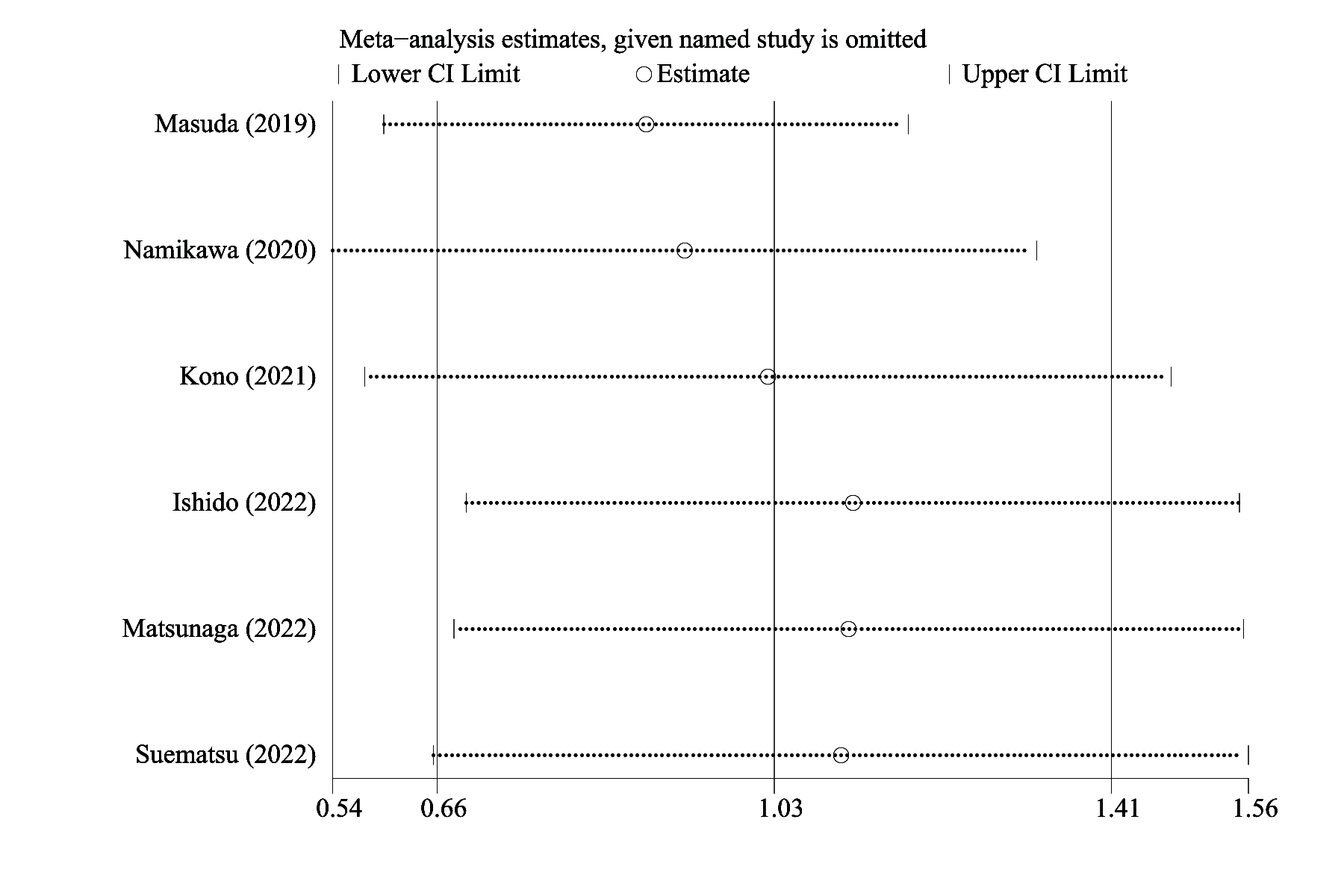

Supplement: Supplementary Figure 6 — Sensitivity analyses for the pooled MSR of PFS, conducted by repeating the analyses with exclusion of studies one by one. [file Image6.tif]
