# Supplementary material for: Association between immune-related adverse events and the prognosis of patients with gastric cancer treated with nivolumab: a meta-analysis
Source: Front Oncol. 2024 Sep 5;14:1408755. doi: 10.3389/fonc.2024.1408755 (PMC11410568; doi:10.3389/fonc.2024.1408755)
Supplement: Supplementary file 7 [file Table1.docx]

**Supplementary Table 1.** Test of publication bias for primary outcomes assessed with Begg’s and Egger’s tests.

| Outcomes | No. of studies | No. of patients | Test | P-value |
| --- | --- | --- | --- | --- |
| HR for OS | 6 | 393 | Begg’s | ＞0.05 |
|  |  |  | Egger’s | ＞0.05 |
| MSR for OS | 5 | 341 | Begg’s | ＞0.05 |
|  |  |  | Egger’s | ＞0.05 |
| HR for PFS | 6 | 393 | Begg’s | **＜0.05** |
|  |  |  | Egger’s | **＜0.05** |
| MSR for PFS | 6 | 393 | Begg’s | ＞0.05 |
|  |  |  | Egger’s | ＞0.05 |

AE, adverse event; HR, hazard ratio; IRAES, immune-related adverse events; MSR, median survival ratio; OS, overall survival; PFS, progression-free survival.

**Supplementary Table 2.** Results of meta-regression models.

| Outcomes | Characteristics | No. of studies | No. of patients | P-value |
| --- | --- | --- | --- | --- |
| HR for OS | Median follow-up | 5 | 341 | 0.066 |
|  | Median time to onset of irAEs | 3 | 176 | 0.195 |
| MSR for OS | Median follow-up | 5 | 341 | 0.743 |
|  | Median time to onset of irAEs | 3 | 176 | 0.105 |
| HR for PFS | Median follow-up | 5 | 341 | 0.461 |
|  | Median time to onset of irAEs | 3 | 176 | 0.784 |
| MSR for PFS | Median follow-up | 5 | 341 | 0.162 |
|  | Median time to onset of irAEs | 3 | 176 | 0.955 |

AE, adverse event; HR, hazard ratio; IRAES, immune-related adverse events; MSR, median survival ratio; OS, overall survival; PFS, progression-free survival.
